# Second harmonic imaging of membrane potentials: Comparing water and chromophores as probes

**DOI:** 10.1016/j.bpj.2026.06.014

**Published:** 2026-06-11

**Authors:** Iwona Swiderska, Seonwoo Lee, Mischa Flór, Sylvie Roke

**Affiliations:** 1 Laboratory for Fundamental BioPhotonics (LBP), Institute of Bio-engineering (IBI), and Institute of Materials Science (IMX), School of Engineering (STI), and Lausanne Centre for Ultrafast Science (LACUS), École Polytechnique Fédérale de Lausanne (EPFL), Lausanne, Switzerland

## Abstract

The electrostatic potential of lipid membranes is an important metric to gain insight into the physiological state of cells. Second harmonic (SH) imaging has emerged as a powerful tool to perform *in situ* and *in vivo* electrostatic potential measurements, either using targeted probes or label-free by using water as a probe. In this work, we compare both modalities, by performing SH imaging on freestanding lipid membranes in aqueous solutions. Correlating the SH intensity to an applied external potential bias, the resonant voltage probe and the water response display distinctly different responses, which can be traced back to different nonlinear optical mechanisms. These mechanisms ensure that the water imaging provides a clean readout of the transmembrane potential, while the chromophore emission reports on the surface potential in the leaflet where it resides, together with differences in chromophore density, structure, and tilt angles. This means that, while both methods may show similar results, they cannot be compared directly. A combination of both methods is suggested as a useful strategy as the water response is accurate but currently limited to sub-second temporal resolution, while the resonant probe permits a sub-millisecond temporal resolution but is hampered by convoluted structural effects and chemical modification.

## Significance

Membrane potentials regulate essential biological processes including neuronal signaling, ion transport, and membrane permeability, motivating the development of quantitative optical voltage imaging methods. By directly comparing nonresonant second harmonic (SH) imaging from interfacial water and resonant SH imaging of voltage-sensitive fluorophores on the same freestanding lipid membranes, we show that the two approaches report on fundamentally different physical quantities. SH water imaging provides a direct, noninvasive readout of transmembrane potential fluctuations through interfacial water orientation, whereas voltage-sensitive dye FM4-64 imaging mainly reflects chromophore density, orientation, and variations in lipid structure within a single leaflet, making deconvolution of electrostatic and structural contributions nontrivial. These results clarify important limitations in the interpretation and comparison of SH voltage imaging measurements.

## Introduction

Voltage imaging has become an indispensable tool for investigating the electrical activity of excitable cells and tissues, which is critical for understanding processes such as neuronal signaling, muscle contraction, and cardiac function. To improve upon the crude spatial resolution and invasiveness of standard patch-clamp measurements^1^^,^^2^^,^^3^ and the indirectness of calcium imaging,^4^^,^^5^^,^^6^ different approaches have been developed to visualize membrane potentials in real time with high temporal and spatial resolution.^7^

**Figure 1:**
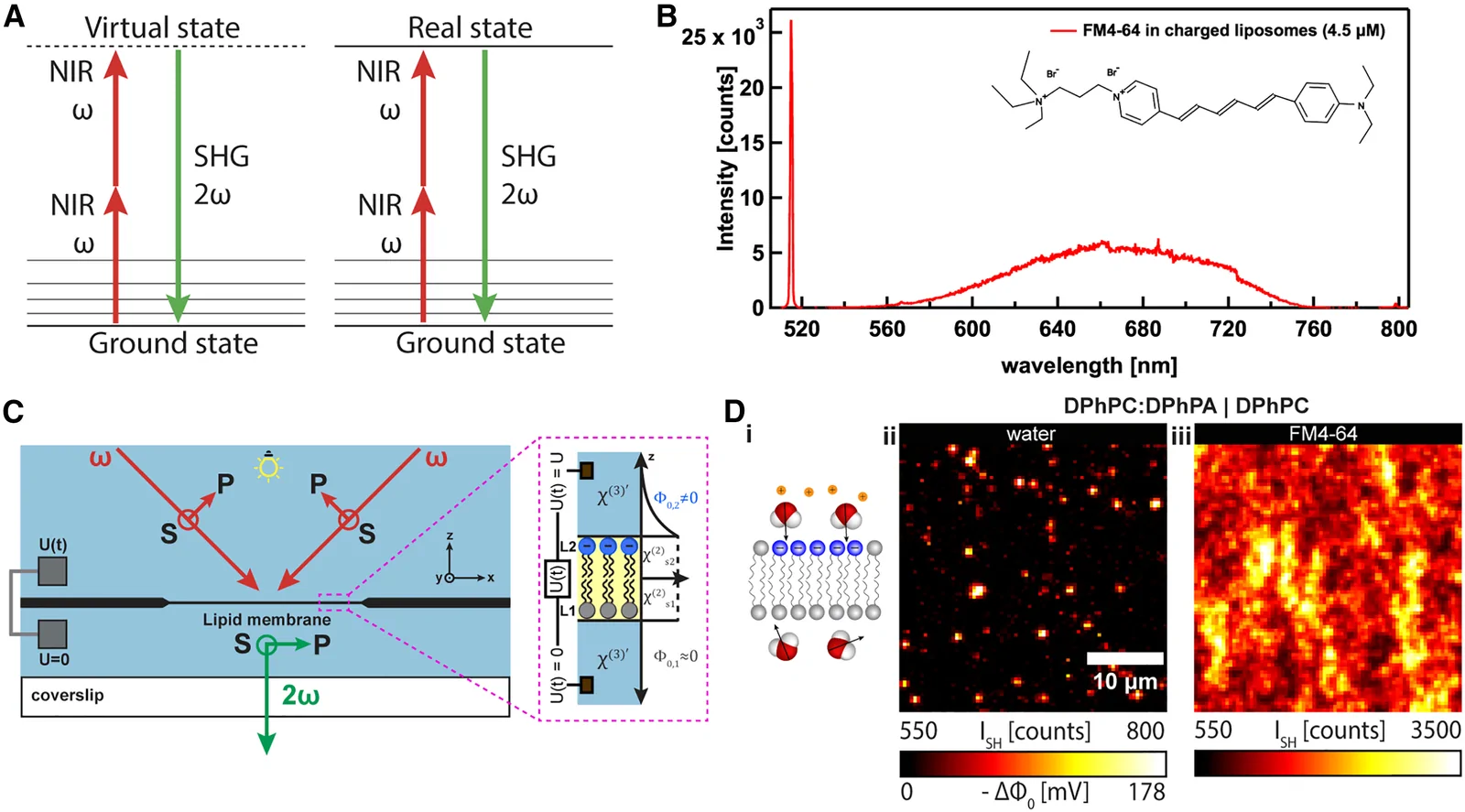
Second harmonic generation with water and FM4-64 dye as a contrast source for FLM imaging. (A) Schematic illustration of the SH process for nonresonant (water) and resonant (FM4-64) cases. (B) Emission spectrum of a POPS liposome solution containing 4.5 μM of FM4-64. (C) Schematic illustration of the SH imaging geometry of the FLMs. Two beams (200 kHz, 190 fs, 1,030 nm) are overlapped in space and time on the lipid membrane. The generated SH photons (515 nm) are collected in the direction of the surface normal. P and S indicate the P-polarized (parallel) and S-polarized (perpendicular) light, respectively.^18^ The zoomed insert shows a schematic illustration of the membrane with one neutral (L1) and one charged (L2) leaflet, the external bias *U*, the susceptibility (*χ*^(2)^ and $\chi^{{\left(3\right)}^{\prime }}$), and surface potential *Φ*_0_. (D) SH images (1-s acquisition time) of an asymmetric membrane composed of 70:30 mol % DPhPC:DPhPA for the top leaflet and DPhPC for the bottom leaflet surrounded by aqueous solution. The scale bar (10 μm) is the same for both images, comparing SH water imaging (ii) and resonant SH chromophore imaging (iii). (i) Illustration of water dipoles oriented by the electric field near the membrane surface, which provides contrast for membrane potential imaging in the case of a pristine membrane. (ii) SH water image obtained when the membrane was in contact with a 165 μM KCl solution. Oriented water molecules generate SH photons, responsible for the image contrast. (iii) SH chromophore image obtained with the top leaflet in contact with a 165 μM KCl solution that also contained 10 μM FM4-64, whereas the bottom leaflet was in contact with a 165 μM KCl solution. The image contrast is here dominated by the resonant FM4-64 SH photons. Note that fluctuations due to noise for the water imaging in (ii) amount to 10 counts, as determined from incoherent SHS from water. Fluctuations due to noise for the fluorophore imaging in (iii) amount to 5 counts, as determined from incoherent SHS from fluorophore containing solutions.

Second harmonic (SH) microscopy^8^^,^^9^^,^^10^^,^^11^^,^^12^^,^^13^^,^^14^^,^^15^^,^^16^ has emerged as a powerful technique for membrane voltage imaging due to its innate interfacial specificity. In a SH experiment, the simultaneous interaction of two photons with frequency ω creates charge oscillations (the second-order polarization, **P**^(2)^ (2ω)) in a material that can occur at a frequency of 2ω. SH emission occurs when the second-order polarization is created within anisotropic molecules arranged in a nonisotropic structure.^17^ The energy level scheme is illustrated in Figure 1A, comparing nonresonant (label-free) and resonant (labeled) SH generation. A surface intrinsically lacks isotropy in the normal direction, and therefore, SH generation can be used to selectively probe interfaces.^19^^,^^20^^,^^21^^,^^22^^,^^23^^,^^24^^,^^25^^,^^26^ There is also an intrinsic sensitivity to electrostatic fields that either emerge from the interface or are externally applied. Originally dubbed “E-FISH” for electric-field-enhanced SH generation,^27^^,^^28^ this method has been used to probe the surface potential (Φ_0_) with the aid of voltage-sensitive chromophores that insert in membranes. The surface potential Φ_0_ is defined as the integral of the residual electric field (*E*_*DC*_) from the bulk up to the surface ($\Phi_{0}=\int_{0}^{+\infty }E_{DC}\left(z\right)\;dz\text{,}$ with *z* the direction of the surface normal and the surface being located at z = 0). These fluorescent dyes report on changes in the surface potential of a membrane leaflet and have largely been explained in terms of an electro-optic mechanism in studies of vesicles and plant and mammalian cells.^12^^,^^29^^,^^30^^,^^31^^,^^32^^,^^33^^,^^34^ Typical integration times per image in a wide-field femtosecond excitation are on the order of a millisecond,^35^ or less.

Recently, SH water imaging^36^ was demonstrated, in which water molecules are used as intrinsic voltage reporters.^37^ This strategy avoids phototoxicity effects and chromophore-induced structural changes. SH water imaging capitalizes on electric-field-water dipole interactions that occur at the membrane surface and in the electric double layer adjacent to the surface, offering a direct method to extract membrane potential maps with a temporal resolution down to ∼100 ms.^38^^,^^39^ These maps can be converted into reaction constants and free energy landscapes.^40^ SH water imaging has been used to measure surface potential maps of the inner and outer walls of a borosilicate microcapillary surface, which were converted into pK_a_ maps of the glass.^41^ Transmembrane potential maps and videos were also generated from freestanding lipid membranes (FLMs)^38^^,^^40^^,^^42^^,^^43^^,^^44^^,^^45^ and giant unilamellar vesicles (GUVs),^46^^,^^47^ as well as from the membranes of cortical neurons *in vitro*.^48^ All charged interfaces displayed membrane potential fluctuations. The source of these fluctuations lies in the fact that interfacial surface chemistry is spatially varying and that transport of chemicals in the electric double layer adjacent to the interface is not a diffusion-limited motion but rather determined by jamming, electrostatic surface field gradients, and nanoscale fluctuations, which are also found in concentrated electrolyte solutions.^49^^,^^50^^,^^51^ The spatiotemporal variation of the charged state of lipids and ions along lipid leaflets induces transient electrostatic fields, which result in the observed membrane potential fluctuations.^39^^,^^46^ These fields have been shown to lower the energetic barrier for the formation of transient nanopores^46^^,^^47^ and water wires/needles.^45^

Here, we compare SH water imaging to resonant SH voltage imaging using an amphiphilic dye, FM4-64, on a freestanding lipid membrane system. The response to an external electrostatic field is different for both methods, which is explained in terms of a difference in the dominant nonlinear optical parameters: labeled surface voltage probing primarily relies on the second-order surface susceptibility of the probe, while water imaging is dominated by the effective third-order susceptibility of the water. Both methods reveal membrane potential fluctuations, but the apparent spatial and temporal behavior is different, which arises from the same difference in response mechanism. In the case of water imaging, only transmembrane potential fluctuations are recorded, while for chromophore imaging, the surface potential information of the leaflet that contains the chromophore is mixed up with differences in the localized interfacial number density and orientational distribution function of the chromophore.

## Materials and methods

### Chemicals and cleaning procedures

1,2-Diphytanoyl-sn-glaycero-3-phosphocholine (DPhPC), 1,2-diphytanoyl-sn-glycero-3-phosphate (DPhPA), 1-palmitoyl-2-oleoyl-sn-glycero-3-phospho-L-serine (POPS), in powder form (>99%, Avanti Polar Lipids, Alabama, USA), hexadecane (C_16_H_34_, 99.8%, Sigma-Aldrich), hexane (C_6_H_14_, 99%, Sigma-Aldrich), chloroform (CHCl_3_, 99.8%, Merck), hydrogen peroxide (H_2_O_2_, 30%–32%, Reactolab SA), sulfuric acid (H_2_SO_4_, 95%–97%, ISO, Merck), KCl (99.999%, Aros), agar ((C_12_H_18_O_9_)_n_, >99%, Sigma-Aldrich), and *N*-(3-Triethylammoniumpropyl)-4-(6-(4-(Diethylamino) Phenyl) Hexatrienyl) Pyridinium Dibromide (FM4-64 dye, Thermo Fisher Scientific, Figure 1B inset) were used as received. The chemical structures of the dye and lipids are shown in Figure S1. All aqueous solutions were made with ultrapure water (H_2_O, Milli-Q UF plus, Millipore), which had an electrical resistance of 18.2 MΩ·cm. All aqueous solutions were filtered with 0.1-μm Millex filters. For each experiment, the coverslips were taken freshly from the packaging and were pre-cleaned by soaking them thoroughly in piranha solution (a 1:3 mixture of 30%–32% H_2_O_2_: 95%–97% H_2_SO_4_) for 20 min, after which they were thoroughly rinsed with ultrapure water. For lipid membrane formation, 13 mM lipid-chloroform solutions were used, which is sufficient to make a well-packed lipid monolayer on an air/water interface. Lipid mixtures were prepared by mixing the lipid-containing solutions to the desired molar ratios. To introduce the FM4-64 dye into the membrane, a small quantity of FM4-64 solution in water was pipetted into the top chamber solution until the final concentration of 10 μM.

### Formation of freestanding horizontal planar lipid bilayers

Freestanding horizontal planar lipid membranes were formed using the procedure of Montal and Müller,^52^ and modifications were applied to form horizontal instead of vertical membranes.^53^ Two separate lipid monolayers were formed on two air/water interfaces that are separated by a 25-μm-thick Teflon film that has an ∼100-μm-diameter aperture in the center. The lipid monolayers were brought into contact by rotating the Teflon film through the interfaces, and a bilayer was formed in the center. The process is schematically shown in Figure S2A. The composition of the leaflets and the aqueous solution that surround the bilayer were controllable *in situ*. To facilitate membrane formation and the transition between the ∼4-nm-thick lipid bilayer and the 25-μm-thick Teflon film, a 99.5:0.5 vol % mixture of hexane and hexadecane coating was applied to the edge of the aperture of the film. When the lipid bilayer was formed, a torus of hexadecane (after hexane evaporation) was formed close to the edge of the aperture and was visible as a Newton diffraction ring, as shown in Figure S2B. Considering a diffusion constant of 1.8 ± 1.0 μm^2^/s for hexadecane and the diameter of membranes, we waited ∼10^3^ seconds for hexadecane to move to the reservoir at the edge before the electrical characterization and SH measurements.

### Wide-field high-throughput second harmonic imaging

Two beams from a Yb:KGW femtosecond laser (200 kHz repetition rate, 190 fs pulses, 1,030 nm wavelength, Light Conversion Ltd) were incident under a 90° angle, each making a 45° angle with the surface normal (Figure 1C). Each beam was loosely focused using f = 20 cm doublet lens (B coating, Thorlabs) to make an ∼150-μm-diameter excitation area. The polarization of each beam was controlled using a linear polarizer (Glan-Taylor polarizer, GT10-B, Thorlabs) and a zero-order λ/2 wave plate (WPH05M-1030, Thorlabs). The laser power for each arm was set to 160 mW. The phase-matched SH photons were collected by a 50× objective lens (Plan Apo NIR HR Infinity-Corrected Objective, 0.65 NA; Mitutoyo), collimated with a 18-cm tube lens (MT-L; Mitutuyo) and captured by an intensified electronically amplified CCD camera (EM-ICCD, PIMax4; Princeton Instruments). A 400-mm meniscus lens was placed behind the objective lens to remove spherical aberrations induced by the cover slip. The fundamental beam and potential two-photon excited fluorescence (2PF) was blocked using a 900-nm short-pass filter (FES0900; Thorlabs) and a 515-nm bandpass filter (10 nm bandwidth, FL514.5–10). Both filters were placed in the detection path. A λ/2 wave plate and a Glan-Taylor prism were used to control the polarization of the generated SH light. All images were measured with the beams P-polarized, parallel to the plane of incidence. The acquisition time of individual frames was 1 s.

### Electrical characterization of the FLMs

The presence of a bilayer was confirmed with white light imaging (Figure S2B) and by electrical measurements of the specific capacitance, C_m_ > 0.7 μF·cm^−2^, and specific resistance, R_m_ ∼10^7^ Ω·cm^2^. Theoretical estimates of the specific capacitance for solvent-free bilayers range from 0.75 to 0.81 μF/cm^2^.^54^ Since we measured values in this range, it is reasonable to conclude that our membranes are free of hexadecane. For membrane bias response measurements, agar salt bridges were used to provide the stable junction potential of electrodes in contact with solutions and to avoid dye contamination of the electrodes. To prepare agar salt bridges, a mixture of 3% agar (w/v) in a 3 M KCl solution was slowly heated up to 150°C while stirring until the agar was completely dissolved. Polypropylene pipet tips were then immersed in the solution, and the liquid agar was drawn into the tips via a capillary force. The tips were then cooled down to 4°C to solidify the aqueous agar. For each measurement, the freshly prepared tips were taken and backfilled with 3 M KCl solutions. Ag/AgCl electrodes that were connected to a patch-clamp amplifier (EPC-10, HEKA Electronics, Germany) were inserted into the tips and were placed on both sides of the membrane. The current response of the lipid membrane to a given external bias was measured with a sampling frequency of 0.5 kHz. For the recording, a 4-pole Bessel filter with a frequency of 0.1 kHz was used.

## Results and discussion

### Second harmonic imaging from a freestanding lipid bilayer

We compare SH voltage imaging using water and the voltage-sensitive probe FM4-64 at a bulk concentration of 4.5 μM. Figure 1B shows the structure of FM4-64 together with an emission spectrum obtained from an aqueous solution of chromophore-containing liposomes. The interfacial water/chromophores are imaged on FLMs formed in an ∼100-μm-diameter circular aperture in a 25-μm-thick Teflon film, following the Montal-Müller technique (see materials and methods^52^). The asymmetric FLMs, composed of 70:30 mole% DPhPC:DPhPA (DPhPC: 1,2-diphytanoyl-sn-glaycero-3-phosphocholine; DPhPA: 1,2-diphytanoyl-sn-glycero-3-phosphate) for the top leaflet and DPhPC for the bottom leaflet, are SH imaged using a homebuilt medium repetition rate, wide-field SH microscope.^36^^,^^38^^,^^41^^,^^42^^,^^44^ The microscope consists of two laser beams (200 kHz, 190 fs, 1,030 nm), which are loosely focused (excitation area of ∼150 μm) on the membrane plane at an incident angle of 45°. The SH photons at 515 nm are generated in the phase-matched forward direction (along the surface normal). The geometry of the SH imaging, together with the polarization states of the electromagnetic fields, is shown in Figure 1C.

Figure 1D shows averaged SH images (1 frame/s, 20 frames) of two asymmetric bilayers composed of 70:30 mole% DPhPC:DPhPA (top leaflet) and DPhPC (bottom leaflet), surrounded by aqueous solutions. Both leaflets are in contact with a 165 μM KCl solution. The SH image (ii) labeled “water” shows SH water imaging, and image (iii) labeled “FM4-64” shows SH FM4-64 imaging of the same membrane. Image (iii) reports on SH photons emitted from FM4-64 chromophores that are inserted in the top leaflet of the bilayer. The SH water image (ii) reports on SH emission from water molecules on both leaflets: in principle each interfacial water molecule can emit SH photons, but destructive interference from water that has mirrored symmetry on the opposing leaflet ensures that much of this is canceled out. Because the membrane is asymmetric, with only one side of the membrane being charged, an electrostatic field in the direction of the surface normal is present only on the top leaflet. This field interacts with dipolar water molecules that become partially aligned with it. As these aligned water molecules are present only on one leaflet, they are responsible for the generated SH photons. The emitted SH photons report on the magnitude of the surface electrostatic field. This is illustrated in Figure 1D (i).

While both images (ii) and (iii) display intensity variations, indicative of surface potential fluctuations, they look different. These differences will be investigated next by considering first the emission mechanism and then the differences in SH emission as a function of applied external bias.

### Water versus FM4-64 as voltage probes

#### Water

Surface potential (Φ_0_) information derives from the proportionality of the emitted SH field to the induced nonlinear optical polarization (*P*^(*NL*)^ (2*ω*)). *P*^(*NL*)^ (2*ω*) depends on the incoming electromagnetic fields (*E*_1_(*ω*),*E*_2_(*ω*)) from the light source having frequency *ω* and *E*_*DC*_(*z*), the electrostatic field in the aqueous phase that emerges from the charged surface (Figure 1C). It is this field that relates to the electrostatic surface potential Φ_0_, since $E_{DC}\left(z\right)=-\frac{d}{dz}\Phi \left(z\right)$), and the expressions for *P*^(*NL*)^ (2*ω*) and the emitted intensity *I*(2*ω*) become the following^38^^,^^45^:

(1) $$P^{\left(NL\right)}\left(2\omega \right)=\epsilon_{0}\left(\chi_{s}^{\left(2\right)}:E_{1}\left(\omega \right)E_{2}\left(\omega \right)+\chi^{{\left(3\right)}^{\prime }}\vdots \int_{0}^{+\infty }E_{1}\left(\omega ,z\right)E_{2}\left(\omega ,z\right)E_{DC}\left(z\right)\;dz\right)$$

(2) $${I\left(2\omega \right)\propto \left|P^{\left(NL\right)}\left(2\omega \right)\right|}^{2}\text{.}$$

The parameters $\chi_{s}^{\left(2\right)}$, and $\chi^{{\left(3\right)}^{\prime }}$ are the second-order surface susceptibility and the effective third-order susceptibility, respectively. For nonresonant SH imaging of interfacial water, these tensors reduce to scalars ($\chi_{s}^{\left(2\right)}$ and $\chi^{{\left(3\right)}^{\prime }}$).^38^^,^^45^^,^^55^ They report on the orientational distribution of interfacial water in the direction of the surface normal due to chemical interactions ($\chi_{s}^{\left(2\right)}$) and due to electrostatic field-driven interactions ($\chi^{{\left(3\right)}^{\prime }}$), respectively. The interface is assumed to be locally isotropic in the surface plane and nonchiral, so that x = y. In the geometry of Figure 1C, using PPP polarization, with a SH polarization along the x direction (along the surface plane), and the incoming beams polarized in the x-z plane, the x and z containing xxz, xzx components of $\chi_{s}^{\left(2\right)}$ are probed. $\chi_{s,xxz}^{\left(2\right)}=\chi_{s,xzx}^{\left(2\right)}$, because of the off-resonance and nondispersion conditions at the 1,030/515 nm wavelengths, thus making the Kleinman conjecture applicable in this case.^17^ The effective third-order susceptibility elements are determined by the same considerations together with the notion that the electrostatic surface field, which is represented by the last index, appears only in the z-direction. Therefore, the nonzero tensor effective third-order elements for PPP polarization are $\chi_{s,xxzz}^{{\left(3\right)}^{\prime }}=\chi_{s,xzxz}^{{\left(3\right)}^{\prime }}$.

For a lipid membrane with two opposing leaflets (*i* = 1 or 2) and $I\left(\omega \right)={\left|E_{1}\left(\omega \right)\right|}^{2}={\left|E_{2}\left(\omega \right)\right|}^{2}$, the total emitted SH intensity *I*(2*ω*,*x*,*y*) originating from the spatial coordinates *x*,*y* is related to the membrane surface potential on the aqueous side of each leaflet (*Φ*_0,*i*_)^40^:

$$I_{PPP}\left(2\omega ,x,y\right)\propto$$

(3) $${I_{1}\left(\omega ,x,y\right)I_{2}\left(\omega ,x,y\right)\left|\left(\chi_{s1,xxz}^{\left(2\right)}\left(x,y\right)-\chi_{s2,xxz}^{\left(2\right)}\left(x,y\right)\right)+\chi_{xxzz}^{{\left(3\right)}^{\prime }}f_{3}\left(\Phi_{0,1}\left(x,y\right)-\Phi_{0,2}\left(x,y\right)\right)\right|}^{2}$$

Here, *f*_3_ is an interference term involving *E*_1_ and *E*_2_ and determined by the geometry of the experiment.^56^ *f*_3_ = 1 for the transmission experiment used here. The SH intensity variations in the images of Figure 1D are attributed to the transmembrane potential difference Δ*Φ*_0_(*x*,*y*) = *Φ*_0,1_(*x*,*y*)−*Φ*_0,2_(*x*,*y*).

**Figure 2:**
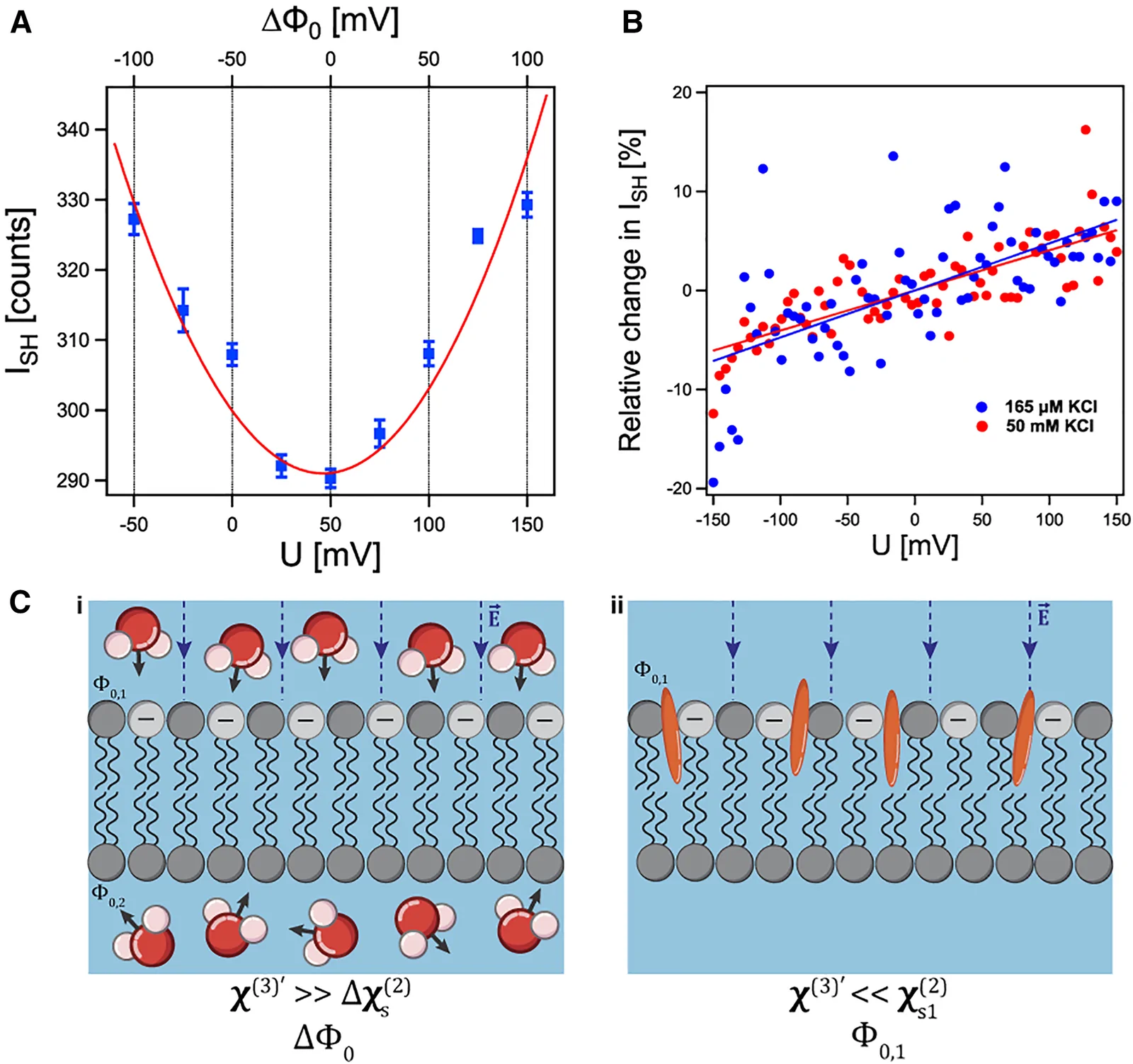
Membrane SH response to an external bias as probed by water versus FM4-64. (A) Spatially averaged SH intensities per pixel as a function of an applied bias (*U*) for an asymmetric membrane composed of 70:30 mol % of DPhPC:DPhPA (top leaflet) and DPhPC (bottom leaflet) and a fit according to Equation 2. (B) Relative change in SH intensity as a function of an applied bias (*U*) for an asymmetric membrane composed of 70:30 mol % of DPhPC:DPhPA (top leaflet) and DPhPC (bottom leaflet) for 165 μM and 50 mM KCl adjacent solutions, where the top leaflet is in contact with 10 μM FM4-46 dye solution. (C) Schematic illustrations of the two different mechanisms that are responsible for the emission of SH photons. (i) Nonresonant SH water imaging and (ii) resonant SH chromophore imaging.

Equation 3 shows that Δ*Φ*_0_(*x*,*y*) is proportional to $\sqrt{I\left(2\omega ,x,y\right)}$. To obtain Δ*Φ*_0_(*x*,*y*) in V, the image-integrated SH intensity is recorded as a function of an external electric bias that is applied across the membrane (*U*). Figure 2A shows the spatially averaged SH intensities per pixel as a function of such a bias (*U*, between −50 and +150 mV) for an asymmetric membrane composed of 70:30 mol % of DPhPC:DPhPA (top leaflet) and DPhPC (bottom leaflet). This is the same membrane as used in Figure 1. The external bias was controlled with a HEKA patch-clamp amplifier (EPC 10; HEKA Elektronik) using two Ag/AgCl electrodes submerged in the opposite solutions (more details in materials and methods), as depicted in Figure 1C. The data have a quadratic/parabolic dependence on the applied bias, and the empirically obtained voltage sensitivity is ∼14%/100 mV. A fit to this data converts the intensity into a membrane potential difference in mV (see Tarun et al.^38^ for details). With $\left|\chi^{{\left(3\right)}^{'}}\right|$ = 10.3 · 10^−22^ m^2^/V^2^ and $\left|{\chi \;}_{s1}^{\left(2\right)}-{\chi \;}_{s2}^{\left(2\right)}\right|$ = 5 · 10^−24^ m^2^/V determined, the transmembrane potential Δ*Φ*_0_(*x*,*y*) of every pixel in the SH image can be computed in mV using Equation 3 and is shown in Figure 1D (ii). The values obtained by this procedure were previously confirmed in three ways: first, the image-averaged membrane potential difference was compared with the averaged membrane potential difference measured by capacitance minimization, a purely electrical recording.^38^ Second, membrane-averaged binding constants of divalent ions (Ca^2+^, Ba^2+^) that complex with certain lipids were computed from the transmembrane potential values,^40^ and the values matched with values obtained by other independent methods. Finally, simulation data matched the experimental results.^40^

#### FM4-64

Figure 2B shows the SH intensity recorded for the same asymmetric membrane with the top leaflet additionally in contact with a 10 μM FM4-64 solution. Two cases of adjacent solutions were measured, 165 μM and 50 mM KCl_(aq)_, and the applied bias ramp ranged from −150 to +150 mV. In this case, the SH intensity shows a linear instead of a quadratic trend. The empirically obtained voltage sensitivity is ∼4.7%/100 mV as determined by the linear fit. This trend agrees with previous recordings,^12^^,^^31^^,^^32^ which suggests a different response mechanism compared with the SH water imaging. Equations 1 and 2 are still valid, but here the SH emission is governed by the resonantly enhanced response of the chromophore, which inserts only in the top leaflet, thereby only probing *Φ*_0,1_(*x*,*y*). The nonzero tensor elements of $\chi_{s}^{\left(2\right)}$ and $\chi^{{\left(3\right)}^{\prime }}$ of the chromophore that is probed in this experiment are the same as for water but originate from geometrical considerations rather than energy level spacing/dispersion. These symmetry considerations are possible even with a resonant SH process because FM4-64 has a rod-like shape (Figure 1B) leading to the nonlinear optical response being dominated by a single hyperpolarizability component having all indices along the molecular axis. In this case, the permutation symmetry is serendipitously recovered even at resonance even though it does not stem from Kleinman’s conjecture.^57^ Therefore,

(4) $$I_{PPP}\left(2\omega ,x,y\right)\propto I_{1}\left(\omega ,x,y\right)I_{2}\left(\omega ,x,y\right){\left|\chi_{s1,xxz}^{\left(2\right)}\left(x,y\right)+\chi_{xxzz}^{{\left(3\right)}^{\prime }}\;f_{3}\left(\Phi_{0,1}\left(x,y\right)\right)\right|}^{2}\text{.}$$

Because the experimental result is a linear curve and not a parabola, in this case $\chi_{s1,xxz}^{\left(2\right)}\gg \chi_{xxzz}^{{\left(3\right)}^{\prime }}$, (instead of $\left|\chi^{{\left(3\right)}^{'}}\right|$ |$\gg |\chi_{s1}^{\left(2\right)}-\chi_{s2}^{\left(2\right)}|$, for water) which transforms Equation 4 into

(5) $$I\left(2\omega ,x,y\right)∼I_{1}\left(\omega ,x,y\right)I_{2}\left(\omega ,x,y\right)\left[{\left(\chi_{s1,xxz}^{\left(2\right)}\left(x,y\right)\right)}^{2}+2{\chi_{s1,xxz}^{\left(2\right)}\left(x,y\right)\chi }_{xxzz}^{{\left(3\right)}^{\prime }}\Phi_{0,1}\left(x,y\right)\right]\text{.}$$

The insensitivity to the ionic strength in the solution (Figure 2B) confirms that $\chi_{s1}^{\left(2\right)}\gg \chi^{{\left(3\right)}^{\prime }}$. Note that in the case of SH water imaging, there is a strong dependence on ionic strength,^38^ caused by the dominance of the $\chi^{{\left(3\right)}^{\prime }}$ term, and its dependence on the ionic screening length in the solution.^56^

The dominance of $\chi_{s1,xxz}^{\left(2\right)}$ complicates matters for obtaining the surface potential. First, Equation 5 is sensitive to the chromophore-containing leaflet only and therefore is sensitive to *Φ*_0,1_(*x*,*y*) and changes therein. Second, the response dictated by the second-order surface susceptibility $\chi_{s1}^{\left(2\right)}$ depends on a number of other parameters that are not only connected to the electro-optic response of the chromophore but also to the chromophore structuring on the interface: the surface number density *N*_*s*_ of the chromophores, its tilt angle *θ* with respect to the surface normal, and its averaged orientational distribution, indicated by ⟨ … ⟩^58^:

(6) $$\begin{array}{c} \chi_{s1}^{\left(2\right)}=\frac{1}{\epsilon_{0}}N_{s}\beta^{\left(2\right)}\left\langle \theta \;\right\rangle \text{.} \end{array}$$

*β*^(2)^ is the second-order hyperpolarizability of the chromophore, which relates to the surface potential through electro-optic coupling, and *ε*_0_ is the vacuum permittivity. Since *β*^(2)^ contribution through $\chi_{s1}^{\left(2\right)}$ is modulated by *θ* and *N*_*s*_ a direct determination of *Φ*_0,1_(*x*,*y*) through Equation 5 is not possible. Also, Equation 6 assumes a homogeneous distribution; if the chromophores adsorb in islands, it would be different and complicate matters more. If one performs a very crude approximation and uses the empirical trend in Figure 2B a calculation of the surface potential can be attempted, but since the SH intensity of the chromophore more strongly depends on its density and tilt angle and therefore differs wildly across the sample and between samples, it is not a reliable conversion (section S2 in the supplemental information describes the result when this is attempted, leading to an unphysical result). Therefore, although both water and FM4-64 are sensitive to electrostatic potentials, there are clear differences in the reporting mechanism and what can be obtained from it. Figure 2C schematically illustrates the two response mechanisms, along with the dominant nonlinear susceptibilities that dictate the SH response.

### Membrane potential fluctuations as seen by water versus FM4-64

**Figure 3:**
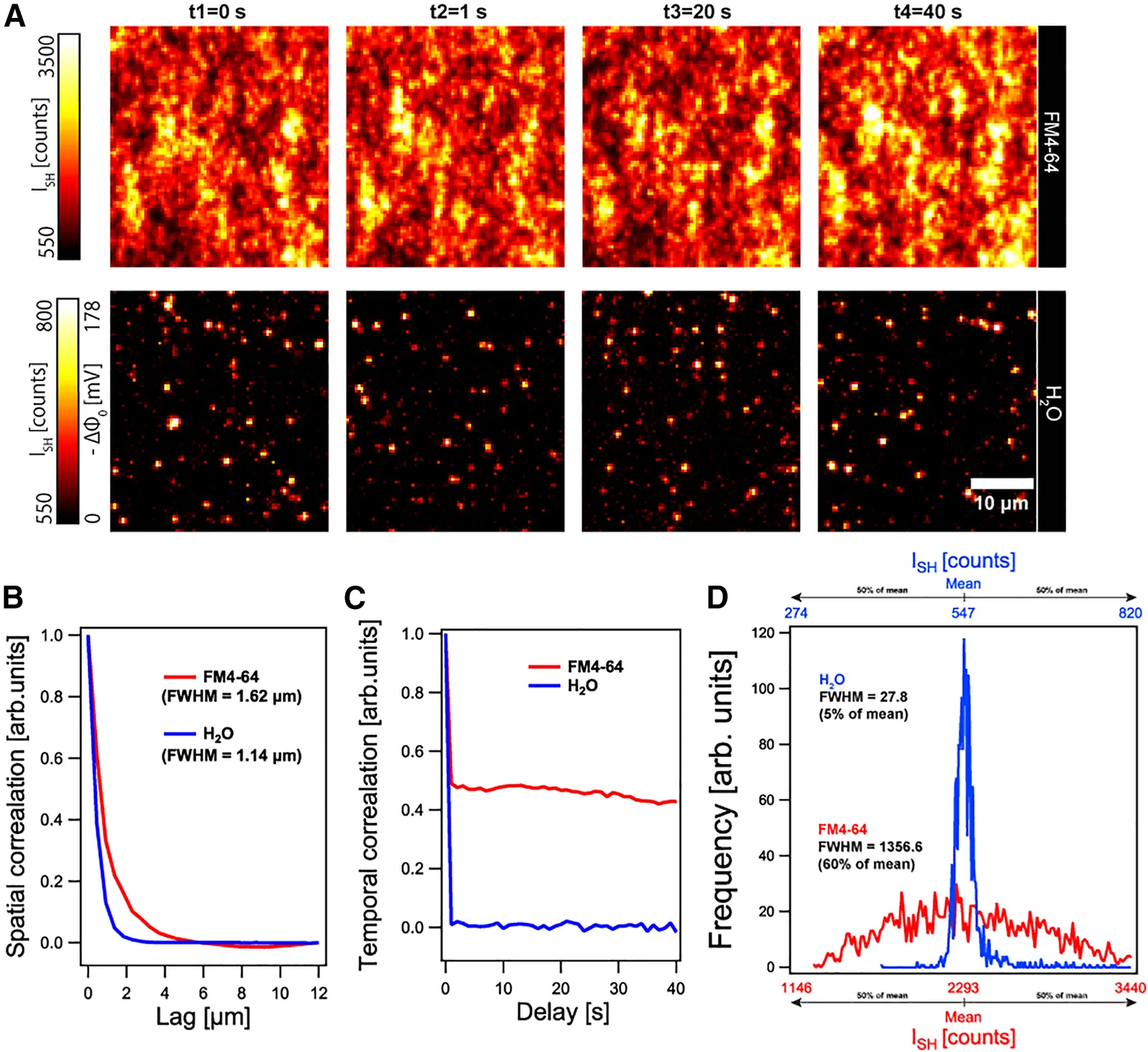
Membrane potential fluctuations as seen by SH water and FM4-64 imaging. Comparison between an asymmetric membrane composed of 70:30 mol % of DPhPC:DPhPA (top leaflet) and DPhPC (bottom leaflet) SH imaged using 10 μM FM4-64 as a probe (top) and water as a probe (bottom). The top and bottom leaflets are in contact with a 165 μM KCl solution. (A) Time series of SH images (1 s each) for both cases. The scale bar (10 μm) is the same for all images. (B) Normalized spatial autocorrelation function (SACF) of the single-frame images (40 frames total, 1-s acquisition time). The FWHM from a Gaussian distribution fit equals 1.14 μm for H_2_O and 1.62 μm for FM4-64. The dye shows domains that are 42% bigger than the water domains. The spatial resolution of the measurement is 390 nm. (C) Normalized temporal autocorrelation function (TACF) over 40 frames (1-s acquisition time). Both imaging modalities show a fast component, shorter than 1 s, most likely arising from electrostatic interactions of water in the electric double layer. FM4-64 SH imaging also shows a slow component, longer than 40 s, likely related to the surface chemistry within the lipid membrane. The temporal resolution of the measurement is 1 s. (D) Histogram of the averaged SH intensity (40 frames, 1-s acquisition time). The horizontal axes are respectively centered at the mean intensity, and they span ±50% of the respective mean. The FWHM from a Gaussian distribution fit equals 5% of mean for H_2_O and 60% of mean for FM4-64, showing larger intensity fluctuations for the membrane with FM4-64.

To determine how both methods report on membrane potential fluctuations, we performed a single frame analysis (1 frame/s) of the membrane system investigated in Figure 1D. Figure 3A shows the spatiotemporal evolution of the domains showing four frames recorded at 0, 1, 20, and 40 s of the acquisition series (the videos are available in Video S1). Comparing both image arrays, the domains imaged by the chromophore are in general bigger than those SH imaged by water. They also appear to persist in the same areas on the SH images over longer timescales when compared with the SH water imaged domains. To characterize this behavior, we performed spatial and temporal autocorrelation function (SACF, TACF) analysis. Figure 3B shows the average normalized SACF of the 40 frames fitted with a Gaussian curve for FM4-64 and water images. The full-width at half maximum (FWHM) of the SACF reports on the characteristic radius of the domains, which is equal to 1.62 μm for FM4-64 and 1.14 μm for water (the spatial resolution of the measurement is 390 nm). The domains in the chromophore SH images are ∼42% bigger than the water domains. As the SH water imaging reports on transmembrane potential differences only, the fluctuations in Δ*Φ*_0_(*x*,*y*) lead to domains with average sizes of 1.14 μm. The added size as seen by the FM4-64 imaging reports on both fluctuations in *Φ*_0,1_(*x*,*y*) as well as correlations in the orientational distribution of FM4-64. The chromophores are orientationally correlated with one another in domains, which is a purely surface-structural phenomenon. Furthermore, unlike the water SH imaging, only one leaflet is probed instead of both, and therefore the cancellation of nonlinear optical responses from opposing leaflets does not occur in the FM4-64 SH imaging experiment.

Video S1. SH imaging video: Water versus FM4-64 dye

The normalized temporal autocorrelation function (TACF) (Figure 3C) was obtained using consecutive time frames (40 frames in total) and reports on the characteristic lifetime of the domains. Both water and FM4-64 membranes present a TACF component that decays faster than the acquisition time of the measurement (1 s). The domains as seen in the SH water imaging are thus not temporally correlated on the timescale of acquisition, and this matches with charge density fluctuations in the electric double layer.^38^ However, when the asymmetric membrane is SH imaged with FM4-64, the domains show an additional TACF component, which persists for longer than 40 s. These correlations also match with surface structuring of the chromophores, as these occur on timescales longer than a few seconds.^59^

Another way of showing the different SH emission mechanisms is demonstrated in Figure 3D, a histogram of the averaged SH intensity taken over 40 frames (1 frame/s) as seen by SH water imaging (blue, top X axis) and SH FM4-64 imaging (red, bottom X axis). The horizontal axes are both centered around the mean values and span 50% of the mean in both directions. The SH intensity distribution in the case of SH water imaging has a FWHM of ∼5% of the mean, which reflects the distribution of membrane potential values. FM4-64 SH imaging shows a SH intensity distribution with a FWHM that is ∼60% of the mean. The fluctuations in intensity are therefore a lot larger when probed with FM4-64 compared with water. This difference again reflects the added influence of surface density and structure/orientational distribution of FM4-64 itself in the membrane.

The differences between membrane potential imaging by means of a targeted chromophore probe, such as FM4-64, and label-free using interfacially oriented water molecules are summarized in Table 1. For both cases the SH optical response can be traced back to the dominant nonlinear susceptibility elements, which are different in both cases, resulting in a parabolic response for water imaging and a linear response in the FM4-64 case. Because the FM4-64 SH response is strongly impacted by its nonlinear surface parameter, i.e., $\chi_{s}^{\left(2\right)}$, it reports mainly on the membrane structure of the leaflet that holds the chromophore. The SH response mechanism is therefore not purely electro-optic but is more strongly linked to the tilt angle, the orientational distribution function, and the surface density (as reported in Pons et al.^12^). FM4-64 shows advantages in terms of attainable temporal resolution due to its resonant enhancement of the intensity. It reports on the leaflet it is adsorbed into and thus reports only on the membrane potential of that leaflet. However, its dual sensitivity to both membrane potential and to slow, heterogeneous chromophore-lipid interactions limits the applicability to extract membrane potential fluctuations. These additional chemical degrees of freedom introduce long-lived spatial variations in $\chi_{s}^{\left(2\right)}$, which broaden the spatiotemporal SHG fluctuations beyond those observed for interfacial water. It is also prudent to take into account potential toxicity and perturbations to the membrane’s intrinsic properties, which influence the applicability of this type of SH imaging.

**Table 1:** Comparison between resonant surface second harmonic imaging using FM4-64 and nonresonant SH water

	FM4-64	H_2_O
Spatial resolution	390 nm	390 nm
Temporal resolution	<1 ms^35^	∼100 ms^38^
Invasive	yes	no
Noise level	0.9% of mean bulk intensity^a^	1.5% of mean bulk intensity^b^
Fluctuations	60% of mean interface intensity	5% of mean interface intensity
SACF	1.62 μm	1.14 μm
TACF	<1 s and >40 s	<1 s
Dominant NLO factor	$\chi_{s1}^{\left(2\right)}$ (FM4-64 in one leaflet)	$\chi^{{\left(3\right)}^{\prime }}$ (H_2_O in EDL)
Voltage sensitivity	4.7%/100 mV linear	14%/100 mV quadratic

a Extracted from Figure S3 in supplemental information.

b Estimated from Figure 2 in supplemental information of Tarun et al.^40^

SH water imaging is a nonresonant process that can be currently measured down to timescales of ∼100 ms, significantly longer integration times, but it reports on the pure transmembrane potential that can be retrieved from the data. In a practical sense, because membrane potential plays a crucial role in understanding a variety of chemical and biophysical processes, a combination of both methods could be ideal. A labeled approach provides easily accessible experimental information, and it can be used in many ways thanks to the widespread availability of useful chromophores. By following up with SH water imaging the qualitative chromophore information can be supplemented by quantitative information that can be used to obtain a different/deeper understanding of the process that is being investigated.

## Conclusion

In summary, we have compared two SH voltage imaging modalities on a freestanding lipid membrane system immersed in an aqueous solution: SH water imaging and SH chromophore imaging using a voltage-sensitive dye. SH water imaging is a nonresonant label-free approach with a current temporal resolution of ∼0.1 s. There is therefore no modification to the system under study. The voltage that is recorded is the transmembrane potential difference, which can be retrieved from recording the SH intensity as a function of external bias. Because of the intrinsic symmetry of a lipid bilayer, $\chi^{{\left(3\right)}^{\prime }}$ is the determining factor and originates from water. The contrast in the SH images derives from water molecules that are preferentially oriented by surface/electric double layer electrostatic fields, which makes it a unique reporter on transmembrane potential changes. Since water is the major compound in any such imaging experiment, $\chi^{{\left(3\right)}^{\prime }}$ does not change significantly with dissolved compounds, creating a method that is universally applicable. The sensitivity to the transmembrane potential is a ∼14% change in intensity over a 100-mV potential change.

SH chromophore imaging is a resonantly enhanced labeled approach with a current temporal resolution of <1 ms.^35^ Phototoxicity and chemical modification of the membrane are issues that need to be accounted for. The voltage that is reported on is the surface potential of the leaflet in which the chromophore is inserted. The SH emission from the chromophores is determined by $\chi_{s1}^{\left(2\right)}$. Therefore, the SH intensity not only reports on the surface potential but also on structural aspects of the membrane leaflet in which the chromophore is adsorbed such as domain formation, molecular tilt, and density fluctuations. Upon applying a bias, the surface potential changes, which leads to a ∼5% change in intensity over a 100-mV potential change.

Comparing SH images of both modalities recorded on the same membrane system shows different behaviors, which are attributed to additional chromophore structuring that ends up in the optical images. Using both methods can allow for a combination of high temporal accuracy (from the chromophore) with accurate quantification of membrane potential changes and related phenomena such as binding constants and free energy differences.

## Data availability

The data that support the findings of this study are available from the corresponding author upon reasonable request.

## Acknowledgments

This work was supported by the Julia Jacobi Foundation, the European Union’s Horizon 2020 research and innovation program under Marie Skłodowska-Curie grant agreement 860592 (H2020-MSCA-ITN, PROTON), and the European Research Council grant agreement no. 951324 (H2020, R2-tension).

## Author contributions

I.S., S.L., and M.F. collected the experimental data. I.S. and S.L. performed reproducibility validations. I.S. and S.L. did the image analysis. I.S., S.L., and S.R. analyzed and interpreted the data. I.S. and S.R. wrote the manuscript. S.R. supervised the work. All authors contributed to the article and approved the submitted version.

## Declaration of interests

The authors declare no competing interests.

## Supplemental information

Document S1. Figures S1–S3 and Sections S1–S3

Document S2. Article plus supplemental information
