# Supplementary material for: Second harmonic imaging of membrane potentials: Comparing water and chromophores as probes
Source: Biophys J. 2026 Jun 11;125(14):3707–17. doi: 10.1016/j.bpj.2026.06.014 (PMC13507475; doi:10.1016/j.bpj.2026.06.014)
Supplement: Supplementary file 1 [file mmc1.pdf]

**Biophysical Journal, Volume *125***

## Supplemental information

# Second harmonic imaging of membrane potentials: Comparing water and chromophores as probes

**Iwona Swiderska, Seonwoo Lee, Mischa Flór, and Sylvie Roke**

# Supporting Information for:

# Second harmonic imaging of membrane potentials: comparing water and chromophores as probes

Iwona Swiderska[1], Seonwoo Lee[1], Mischa Flór[1], Sylvie Roke[1,#]

[1]Laboratory for fundamental BioPhotonics (LBP), Institute of Bio-engineering (IBI), and Institute of Materials Science (IMX), School of Engineering (STI), and Lausanne Centre for Ultrafast Science (LACUS), École Polytechnique Fédérale de Lausanne (EPFL), CH-1015, Lausanne, Switzerland

*[#]To whom correspondence may be addressed. Email: sylvie.roke@epfl.ch*

**Table of Contents:**

S1. Chemicals and bilayer formation.

S2. Surface potential retrieval from FM4-64, neglecting molecular structural effects.

S3. Noise level of FM4-64 imaging.

**List of figures:**

Figure S1. The molecular structures of the lipids and the voltage probe used in this work.

Figure S2. Formation of a lipid bilayer membrane.

Figure S3. Noise level of FM4-64 imaging determined by measuring the incoherent SH intensity of a 165 μM $KCl_{(aq)}$ solution with 10 μM FM4-64 without a membrane.

## S1. Chemicals and bilayer formation

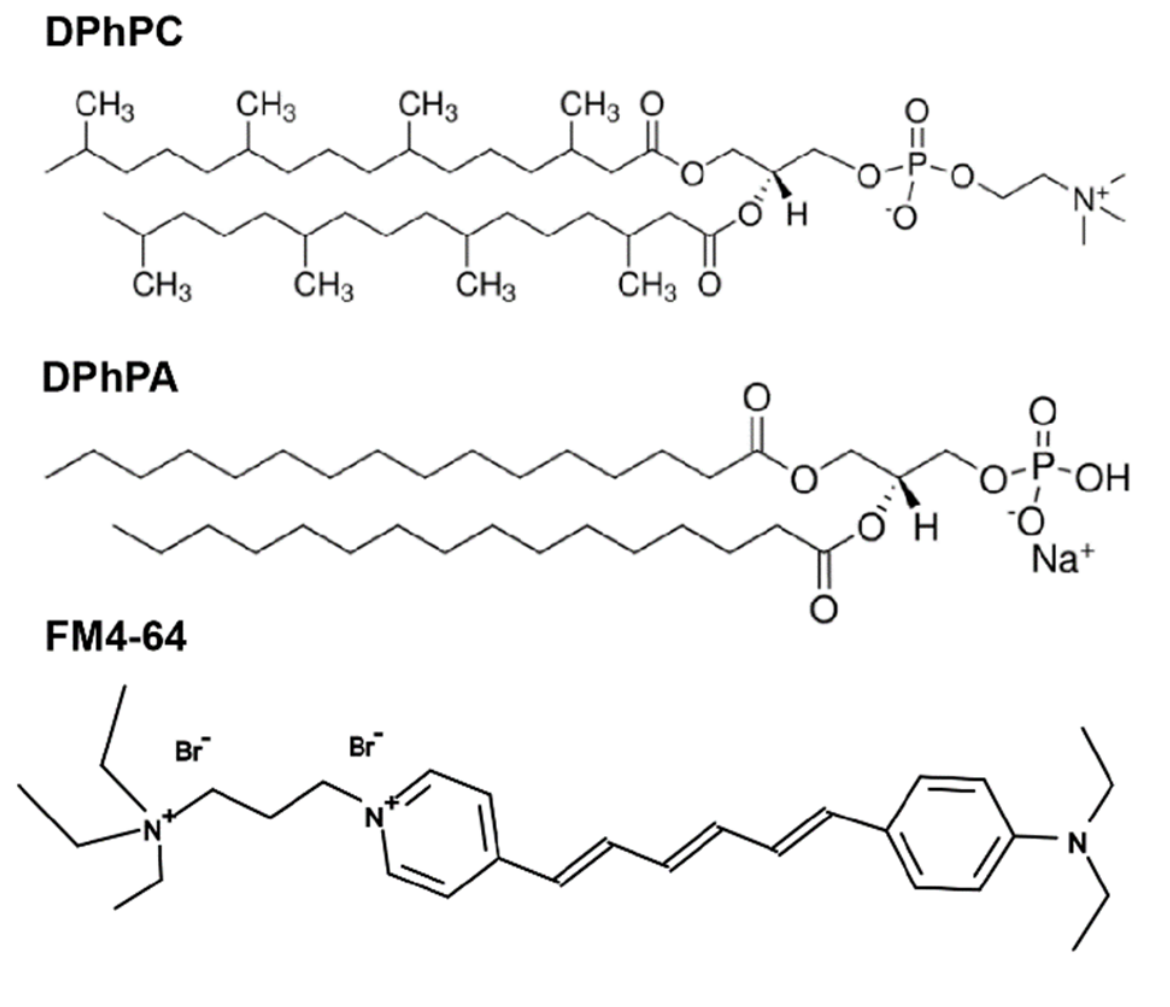


**Figure S1. The molecular structures of the lipids and the voltage probe used in this work.** Fully saturated phospholipids (DPhPC and DPhPA) and the amphiphilic dye (FM4-64).

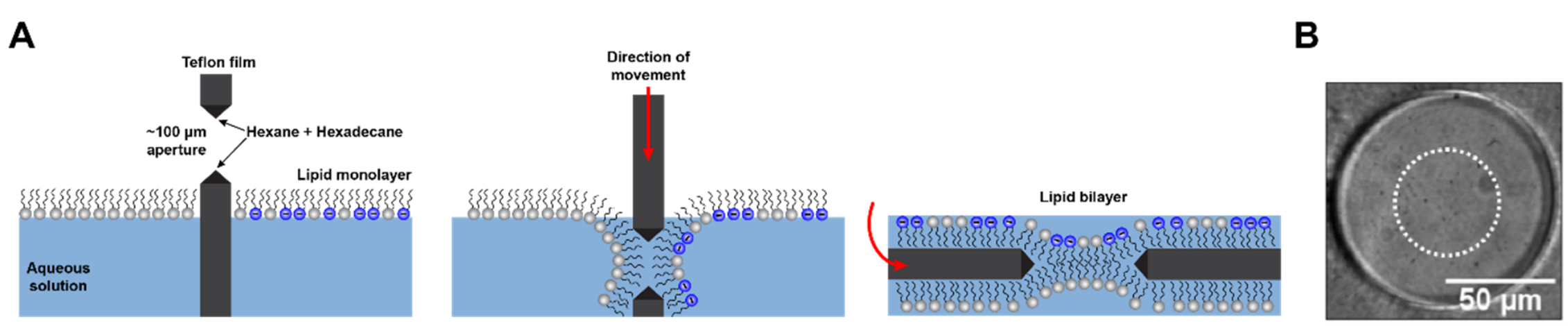


**Figure S2. Formation of a lipid bilayer membrane.** (A) Schematic illustration of the formation of a bilayer membrane. (B) A white-light image of the membrane with a Newton diffraction ring. The central part of the lipid bilayer (white circle) was used to obtain SH images and potential landscapes in this study. Adapted from (1).

## S2. Surface potential retrieval from FM4-64, neglecting molecular structural effects

As discussed in the main text, the retrieval of the surface potential distribution of the membranes from the spatially recorded SH intensity emitted by FM4-64 is impossible because the SH intensity strongly depends on the density and tilt angle in each sample, which vary more than the typical change induced by the electro-optic effect (~4.7 % / 100 mV). If one ignores the dependence of $\chi_{s1}^{(2)}$ on the tilt angle and the density of the chromophores, one can use the empirically measured linear trend between the SH intensity and the applied bias, which results in a function of $I_{SH} = 2654 + 1.078 \cdot U$. In order to compute the change in surface

potential from this expression, it is still necessary to make an approximation on the resting potential of the membrane (potential at 0 mV applied bias), which can be taken from the unperturbed water measurements and set equal to – 50 mV. If one performs such conversion, the scale of the surface potential $\Phi_{0,1}(x, y)$ would range from 0 to – 735 mV with the average value roughly equal to – 72 mV. This range of values is unphysically high as is the temporal persistence, with very high surface potential values being sustained for longer than 40 s. This clearly shows that the SH intensity from the chromophore is dominated by contributions from the surface charge density and the orientational distribution function, which both vary across the membrane.

### S3. Noise level of FM4-64 imaging

In order to measure the noise level for the FM-64 imaging modality, we performed an experiment where 165 µm $KCl_{(aq)}$ solution with 10 µM of FM-64 without forming a membrane was imaged with the same setup and acquisition parameters. Fig S3 shows the time evolution of the incoherent SH intensity from the dye solution recorded this way (A) and the histogram of the SH intensity (B). By fitting the intensity distribution to a Gaussian (μ = 644.8, FWHM = 5.6) we can evaluate the noise level to be equal to 0.9 %.

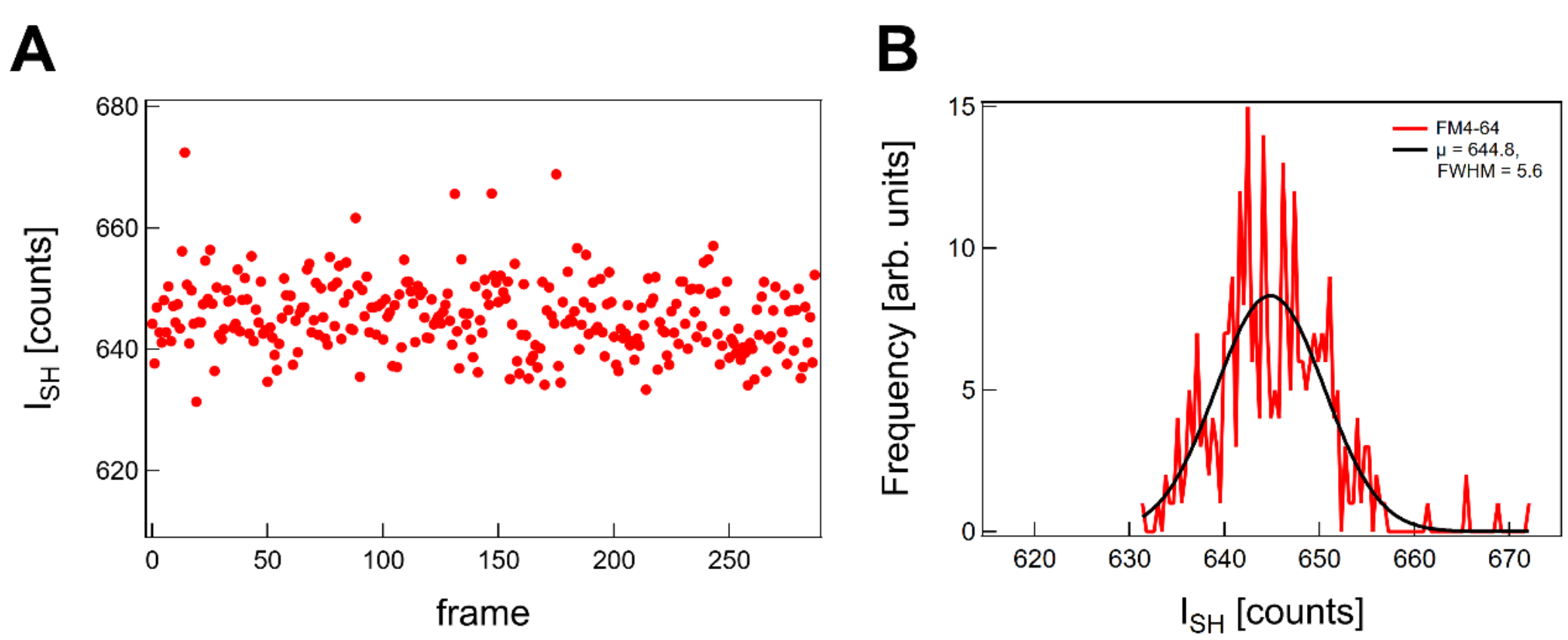


**Figure S3: Noise level of FM4-64 imaging** determined by measuring the incoherent SH intensity of a 165 µM $KCl_{(aq)}$ solution with 10 µM FM4-64 without a membrane**.** (A) Time trace of the SH intensity. (B) Histogram of the SH intensity with a Gaussian fit, μ = 644.8, FWHM = 5.6. The noise level is equal to 0.9 %.

### Supporting References


1. Lee, S., C. S. Poojari, A. Maznichenko, D. Roesel, I. Swiderska, P. Pohl, J. S. Hub, and S. Roke. 2024. Dynamic Second Harmonic Imaging of Proton Translocation Through Water Needles in Lipid Membranes. *Journal of the American Chemical Society*. 146(29):19818-19827, doi: 10.1021/jacs.4c02810, https://doi.org/10.1021/jacs.4c02810.
